# Homologous-adhering/targeting cell membrane- and cell-mediated delivery systems: a cancer-catch-cancer strategy in cancer therapy

**DOI:** 10.1093/rb/rbae135

**Published:** 2024-11-21

**Authors:** Chenguang Liu, Jingjie Gao, Yuying Cheng, Shanshan Zhang, Caiyun Fu

**Affiliations:** Zhejiang Provincial Engineering Research Center of New Technologies and Applications for Targeted Therapy of Major Diseases, College of Life Sciences and Medicine, Zhejiang Sci-Tech University, Hangzhou 310018, P. R. China; Zhejiang Provincial Engineering Research Center of New Technologies and Applications for Targeted Therapy of Major Diseases, College of Life Sciences and Medicine, Zhejiang Sci-Tech University, Hangzhou 310018, P. R. China; Zhejiang Provincial Engineering Research Center of New Technologies and Applications for Targeted Therapy of Major Diseases, College of Life Sciences and Medicine, Zhejiang Sci-Tech University, Hangzhou 310018, P. R. China; Department of Orthopaedics Shanghai Key Laboratory for Prevention and Treatment of Bone and Joint Diseases, Shanghai Institute of Traumatology and Orthopaedics, Ruijin Hospital, Shanghai Jiao Tong University School of Medicine, Shanghai 200025, P. R. China; Zhejiang Provincial Engineering Research Center of New Technologies and Applications for Targeted Therapy of Major Diseases, College of Life Sciences and Medicine, Zhejiang Sci-Tech University, Hangzhou 310018, P. R. China

**Keywords:** homologous targeting, cell membrane, hybrid membrane, delivery system, cancer therapy

## Abstract

Low tumor enrichment remains a serious and urgent problem for drug delivery in cancer therapy. Accurate targeting of tumor sites is still a critical aim in cancer therapy. Though there have been a variety of delivery strategies to improve the tumor targeting and enrichment, biological barriers still cause most delivered guests to fail or be excreted before they work. Recently, cell membrane-based systems have attracted a huge amount of attention due to their advantages such as easy access, good biocompatibility and immune escape, which contribute to their biomimetic structures and specific surface proteins. Furthermore, cancer cell membrane-based delivery systems are referred to as homologous-targeting function in which they exhibit significantly high adhesion and internalization to homologous-type tumor sites or cells even though the exact mechanism is not entirely revealed. Here, we summarize the sources and characterizations of cancer cell membrane systems, including reconstructed single or hybrid membrane-based nano-/microcarriers, as well as engineered cancer cells. Additionally, advanced applications of these cancer cell membrane systems in cancer therapy are categorized and summarized according to the components of membranes. The potential factors related to homologous targeting of cancer cell membrane-based systems are also discussed. By discussing the applications, challenges and opportunities, we expect the cancer cell membrane-based homologous-targeting systems to have a far-reaching development in preclinic or clinics.

## Graphical abstract

**Figure:**
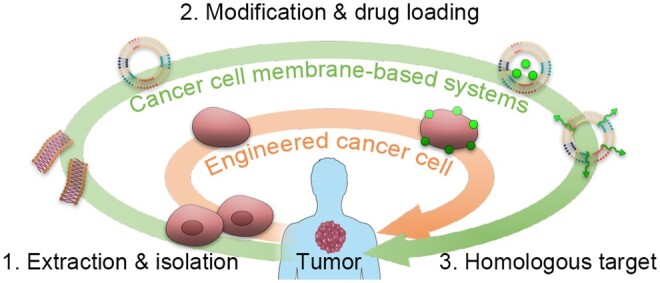

## Introduction

Cancer targeting is a commonplace issue in cancer therapy. One of the important reasons for the failure of cancer therapy is that therapeutic agents (e.g. molecular drug, mRNA, peptide, protein, etc.) are failed to be delivered to the right target, resulting in excessive systemic toxicity [1–4]. A lot of strategies for improving treatment targeting have been developed including the exploitation of new molecular targets, the search for more cancer-selective agents and design of advanced delivery systems [5–8].

Nowadays, drug delivery systems (DDSs) are rapidly evolving in the field of cancer therapy [9–11]. Various DDSs offer different advantages for different therapeutic strategies and administration modalities [12]. For instance, engineered nanoparticles are capable of improving biodistribution and cancer enrichment because of either passive targeting contributed by the enhanced permeability and retention (EPR) effect or active targeting by specific surface modification [13, 14]. Multi-functional hydrogel adheres to specific sites and controllably releases therapeutic agents with specific stimulation (e.g. pH, light, temperature, electric field, etc.) [15–19]. Notably, micro- and nano-systems can be doped into hydrogels or microneedles for multistage-targeted delivery of therapeutic agents.

Turning to active-targeted delivery of micro- and nano-systems, following by classical ligands such as folic acid and hyaluronic acid, aptamers, certain antibodies and peptides are involved [20–22]. Thus, the overexpression or specific expression of corresponding binding ligands at the cancer cell surface endow active targeting capabilities of these ligand-modifying systems to cancer cells with over-expressed folic acid receptor [23], specific antigen [24], epidermal growth factor receptor [25, 26], transferrin receptor [27], etc. However, due to the biological barriers and protein corona, the engineered active-targeting DDSs may be cleared by the system established by Kupffer cells, liver sinusoidal endothelial cells, stellate cells and hepatic cells [28]. Meanwhile, the complicated *in vivo* environment induces the off-target of most DDSs [29]. Wilhelm *et al.* reported that only 0.7% (median) of the administered nanoparticle dose is found to be delivered to tumor sites [30].

Cell-based and cell membrane-based biomimetic DDSs has been gradually emerging in the biomedical field currently [31, 32]. Owing to the presence of surface proteins (e.g. CD44, CD47, etc.) and various adhesion factors (e.g. integrins, selectins, cadherins, etc.), these biomimetic systems can not only send ‘don’t eat me’ signals to macrophages to prolong the blood circulation time but also specifically adhere to tumor tissues to achieve targeted delivery [33, 34]. Biomimetic systems based on erythrocytes and stem cells have a long history of development and are now being pushed into clinical trials [35–37]. Benefiting from the similarity of membrane proteins and membrane components, recently, biomimetic systems constructed from homologous cells have been proven to have homologous-targeting properties. Yu *et al.* reported the nanoparticles coated by chondrocyte membranes can adhere to the cartilage extracellular matrix (ECM) and the internalization of the chondrocyte-membrane-coated nanoparticles was mediated primarily by E-cadherin, clathrin-mediated endocytosis and micropinocytosis [38]. Similarly, cancer cell membranes or engineered cancer cells have been proven homologous-targeting properties and homing capabilities to tumor sites [39, 40]. Therefore, compared with other tumor active-targeting strategies, the homologous-targeting strategy based on cancer cells and cancer cell membranes has the following main advantages: (i) Homologous-targeting systems exhibit highly efficient targeting and immune evasion. Specifically, the homologous-targeting systems, after extracting of cancer cells and constructing the delivery system, directly inherit the homologous targeting and immune escape properties of cancer cells [32], whereas other active-targeting delivery systems often require surface modifications that are susceptible to inactivation under physiological conditions or within the tumor microenvironment. Moreover, cancer cell membranes present multiple composite targeting molecules, unlike the single-target approach commonly used in active-targeting strategies. Single-target systems are more prone to off-target effects as tumors progress and are also more susceptible to immune recognition and clearance after multiple administrations. (ii) Homologous-targeting systems take advantage in biological barrier penetration. Homologous-targeting systems demonstrate better efficacy in crossing biological barriers, such as the colorectal cancer (CRC) barrier and blood–brain barrier [41, 42], thus improving drug bioavailability and reducing associated toxic side effects. (iii) Benefiting from its long circulation in blood and homologous adhesion and targeting, homologous-targeting system can be applied to kill cancer cell in blood, like leukemia, circulating tumor cells (CTCs) [43], and metastatic or migratory cells.

In this review, we focus on not only the source, preparation and verification of homologous-targeting systems but also the advanced applications of various homologous-targeting systems in cancer therapy. In order to combine homologous-targeting capability with enhanced functions, including stability, modifiability and immune response, the cancer cell membrane can hybridize with other membranes derived from stem cells, erythrocyte, leucocytes and bacteria, as well as artificial lipids. Unlike traditional DDSs for cancer therapy, the size of the biomimetic system becomes less consideration. Either micro-/nano-scale cell membrane-coating systems or engineered cancer cells are involved. Moreover, the potential mechanism of homologous targeting and functions of cell adhesion molecules (CAMs) is discussed. Furthermore, the prospects and challenges of cancer cell- or cancer cell membrane-based delivery systems in clinical cancer therapy are accentuated and outlooked.

## Source of homologous-targeting materials and preparation of membrane-based systems

Recently, membrane-based delivery systems have made numerous advances in the field of biomaterials and substance delivery. Membranes of different origins perform different biological functions. For example, cell membranes derived from red blood cells (RBCs), platelets and macrophages have been wildly used to fabricate DDSs for immune escape due to the specific membrane proteins [44–46]. In particular, CD47 expresses highly on the surface of RBCs that is considered to indicate a ‘don’t eat me’ signal and enables the binding to the signal regulatory protein α (SIRPα) on macrophages for the avoidance of phagocytosis [47, 48]. Except for immune escape ability [49], macrophage-membrane-coated nanoparticles have been used for tumor targeting as well due to the overexpression of α_4_ or β_1_ integrins on macrophage cell surface that can bind the vascular cell adhesion molecule-1 (VCAM-1) expressed on the cancer cell surface [50, 51].

Cancer cells also possess a number of immune escape-related molecules on their surface [52]. More importantly, molecules on the surface of cancer cell membranes (e.g. cadherin, galectin-3, etc.) exhibit homologous or heterologous adhesion capabilities, which makes cancer cell membrane-involved delivery systems topical in the active targeting field [53–55]. The source of homologous-targeting materials mainly includes cancer cell membranes and exosomes released from cancer cells. The extraction of membrane materials and biofabrication of homologous-targeting delivery systems are the primary problems in the study of homologous-targeting cancer therapy strategies.

### Cancer cell membrane-based systems

**Figure 1.**
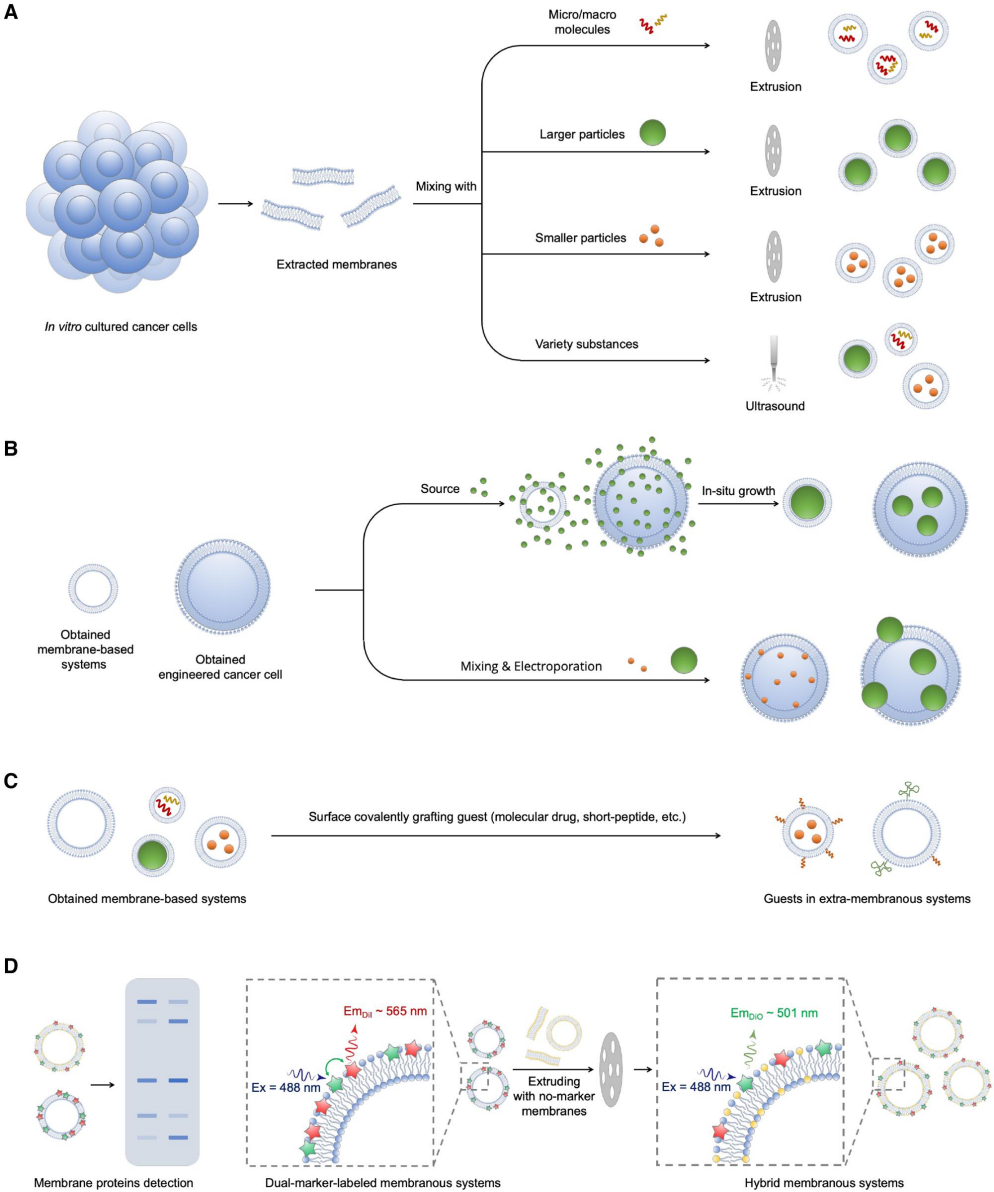
Illustration of preparation of cancer cell membrane-based systems. (**A**) Preparation of membrane-coated systems by membrane extrusion or ultrasonication. The membrane extrusion method is generally considered to provide better regulation of the particle size of the systems and better homogeneity. (**B**) Preparation of membrane-coated or cell-based systems by *in situ* growth or electroporation methods. (**C**) Cancer cell membrane-based systems prepared by grafting some functional substances onto the membrane surface through covalent bonds. These surface-grafted substances can be delivered to homologous tumor sites. (**D**) Components and hybrid detections of obtained membrane systems, including Western blotting and FRET methods.

Cancer cell membrane is an important membrane source for the preparation of homologous-targeting systems. Compared to RBCs, platelets, and immune cells, cancer cells possess the unique ability to proliferate infinitely, making their membrane readily accessible. The method of extracting cancer cell membrane is relatively uniform. As shown in Figure 1, usually collected cancer cells are fragmented by hypotonic or freeze–thaw methods, and cell fractions are subsequently separated by differential centrifugation to obtain cell membrane fragments [56].

#### Preparation methods of cell membrane-based systems

The membrane-based carrier is formed by ultrasound, mechanical membrane extrusion or a combination of the two. The size of the prepared membrane carrier can be easily regulated from 50 to 1000 mm by controlling the ultrasonic time and power, or the pore size of the polycarbonate membrane used in membrane extrusion [52, 57]. It is during the membrane reconstruction process that small-molecule drugs, large-molecule substances or nanoparticles are encapsulated into cell membrane vehicles (Figure 1A). It is noticed that weak interaction forces between nanoparticles and membrane fragments, usually electrostatic adsorption, are relied when preparing a core-shell structure of nanoparticle-membrane by a direct self-assembly method [58]. Therefore, the method of mechanical membrane extrusion is generally used for preparing the core-shell structure of nanoparticle-membrane due to its better versatility [59].

*In situ* growth and electroporation methods are also used to prepare cell membrane-coated nanocomposites (Figure 1B). Zhang *et al.* grew a hydrogel nanoparticle inside a cell membrane-derived vesicle, by which the hydrogel nanoparticle could be completely enclosed and the size of the hydrogel nanoparticle was controlled [60]. Rao *et al.* employed a microfluid chip to achieve an electroporation process [61]. Specifically, Fe_3_O_4_ nanoparticles and as-obtained nano-scale cell membrane-derived vesicles were infused into a microfluidic chip. With the mixing of these two nanoparticles and the effect of electric pulses in an electroporation zone, Fe_3_O_4_ nanoparticles enter into the cell membrane-derived vesicles, resulting in a core-shell constructure [61]. The electroporation method has been proven a higher synthesis efficiency because it can generate a huge number of voids on cell membrane in a very short period of time, thereby promoting the entry of nanoparticles [62].

#### Characterizations of cancer cell membrane-based systems

The structure and size of obtained membrane-based systems can be confirmed by transmission electron microscope and dynamic light scattering [63]. As illustrated in Figure 1D, the obtained membrane-based constructions are usually detected by sodium dodecyl sulfate-polyacrylamide gel electrophoresis to ensure the involvement of certain membrane proteins such as CD44 and CD47 [64]. In the case of hybrid membrane systems, the fluorescence resonance energy transfer (FRET) method is employed to measure the fusion rate [65]. In brief, one type of membrane is labeled with a FRET pair (generally DiO and DiI), and then the labeled membrane fusion with the other type of membrane without being labeled the FRET intensity decrease is measured to dedicate the fusion rate [66].

### Preparation methods of engineered cancer cells and their mechanisms of drug delivery

Cancer cell membrane-based systems are not always reconstituted by membrane fragments, and sometimes an intact cancer cell is also a membrane-based carrier. Compared with cancer cell membrane-based systems, the engineered cancer cells are developed as DDSs independent of the EPR effect. More accurately, cancer cells are not only targeted to cancer cells but also to cancer tissue or paraneoplastic tissue, which is referred to as the homing effect in some cases [67]. Using the homing effect dates back to 2000 when scientists found that neural stem cells (NSCs) ‘surround’ the invading tumor border while ‘chasing down’ infiltrating tumor cells [68]. Sometimes such a method is called ‘hitchhiking’ [69–71]. Later, site-specific tumor cells were also found to tend to target specific tissue sites. For instance, Gu and his team shocked acute myeloid leukemia (AML) cells in liquid nitrogen to eliminate pathogenicity while preserving their major structure and chemotaxis [72].

The methods for loading guest molecules (e.g. small molecules, macromolecules, nanoparticles, etc.) vary depending on their types. Small molecules were initially considered unsuitable for direct cellular delivery, as they typically cross membranes through passive diffusion, making controlled interactions with cells difficult and resulting in unstable loading and potential deficiencies. However, these small-molecule drugs, as well as peptides, proteins or nanoparticles, can form the cell-drug conjugates, which can enhance the functionality of both components, especially in drug delivery and therapy applications [73, 74]. Additionally, molecular drugs such as small molecules, siRNA, peptides and proteins can be loaded into specially functionalized nanoparticles. These nanoparticles, as carriers, can not only attach to the cell membrane *via* electrostatic and hydrophobic interactions, ligand-receptor binding or covalent/non-covalent coupling [75] but also be internalized by cells [76]. Electroporation is another technique used to load nanoparticles into or onto cells, as it creates numerous pores in the cell membrane. For instance, Rao *et al.* used this method to construct platelets loaded with gold nanorods [77].

## Relevant molecules and potential mechanism of homologous targeting

**Figure 2.**
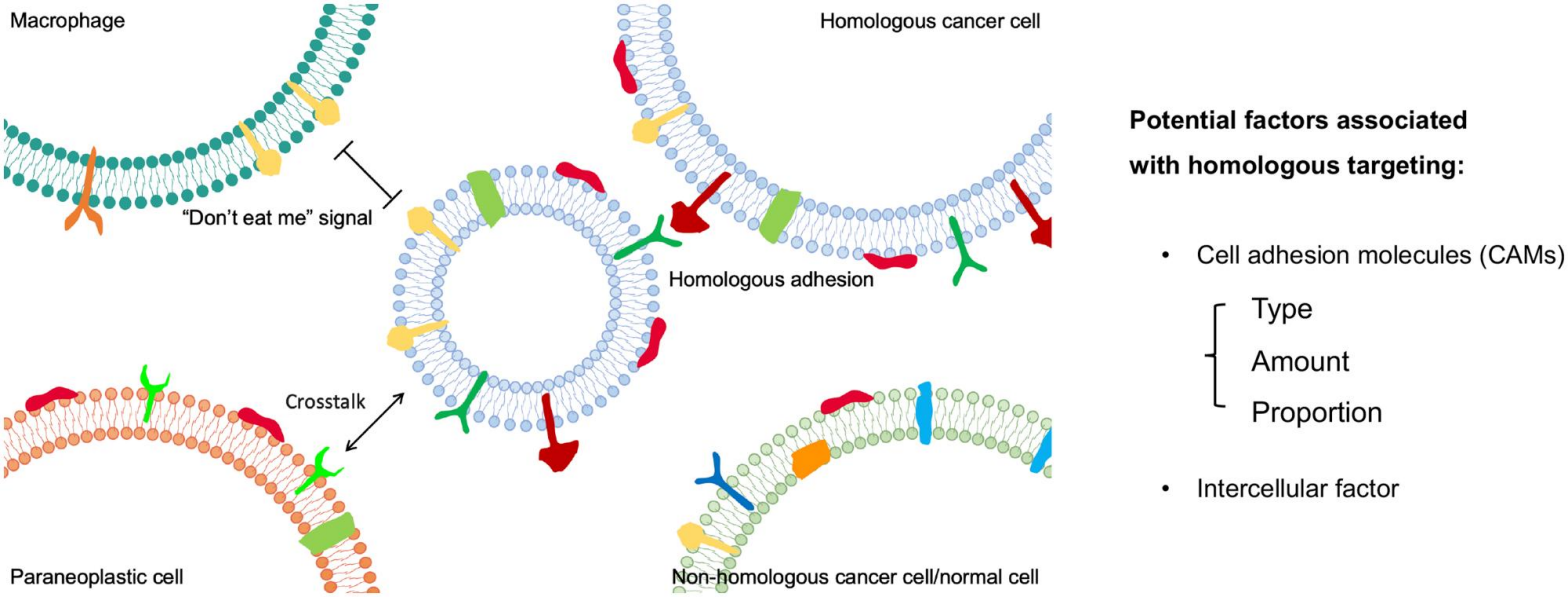
Illustration of homologous targeting of cell membrane-based micro-/nano-size particles.

Homologous targeting is mainly based on the phenomenon of homologous adhesion of cancer cells, which has been observed *in vitro* and *in vivo* [72, 78, 79]. As early as the early 20th century, researchers discovered that cancer cells self-aggregated into spheroids *in vitro* culture [80–82]. In the body, cancer cells can not only circulate in the blood in the form of clusters (e.g. CTCs) and have a higher metastatic capacity than single cells but also tend to homologous adhesion in specific tissues to achieve a favorable environment for proliferation and immune evasion [83–85]. However, the precise mechanism of homologous targeting is not completely clear. At present, homologous targeting is generally considered as the interaction between specific molecules on the surface of homologous cancer cells [86]. As shown in Figure 2, these specific molecules mainly endow cancer cells with the function of self-aggregation, including CAMs and intercellular molecules [86]. More importantly, various cancer cells and cancer cells in different states and conditions exhibit different levels of a certain protein. For instance, small cell hypercalcemic ovarian carcinoma cells demonstrated expression of CD73 and CD105 only when those were co-cultured with mesenchymal stroma/stem cells (MSCs) [87].

CAMs are generally transmembrane receptor proteins and can be divided into four groups: integrins, selectins, cadherins (calcium-dependent adhesion proteins) and members of the immunoglobulin superfamily (IgSF) [88].

Among them, selectors are initially found in platelets, endothelial cells and leukocytes, and thus they are divided into P-, E- and L-selectins depending on the cell type in which they were found [89]. Although selectin has also been found to be highly expressed in some tumor cells [90], to the best of our knowledge, it has not been reported to be used for specific direct tumor targeting and is more often used in an indirect targeting way. For example, by modifying E-selectin on the surface of nanoparticles, Qi *et al.* exploited the affinity binding of E-selectin and leukocytes to achieve the nanoparticles hitchhiking with leukocytes, and thereby enabling indirect tumor targeting [91].

**Figure 3.**
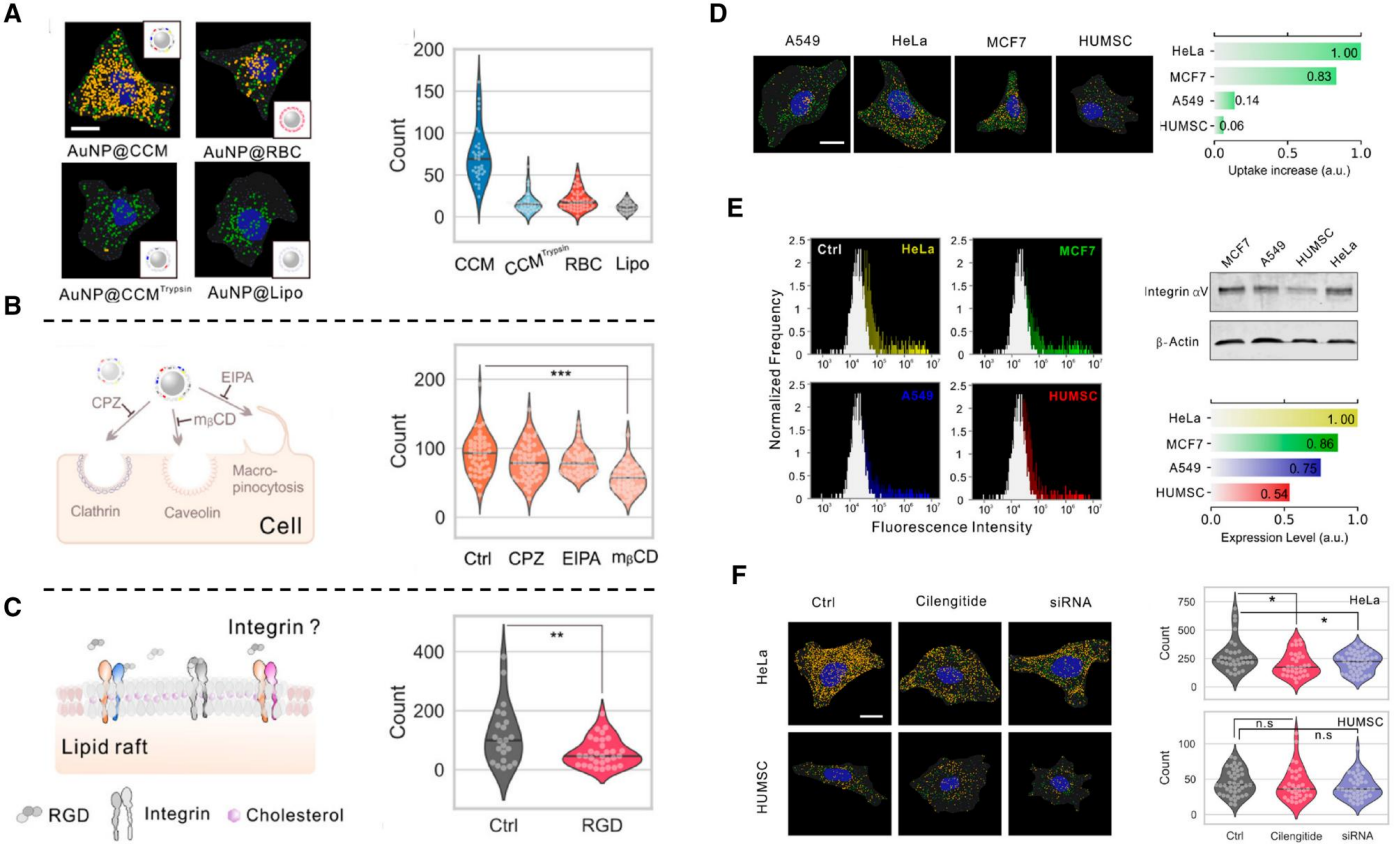
Homologous targeting of AuNP@CCM is regulated by integrin. (**A**) Representative annotated DFM images of HeLa cells treated by AuNPs with different coatings. Scale bar = 10 μm. (**B**) Cellular internalization pathways of AuNP@CCM with treatments of different endocytic inhibitors. (**C**) Illustration of lipid raft and integrin on the cell membrane and count of internalized AuNP@CCMs with/without RGD treatment. (**D**) Annotated DFM images and uptake of A549 cells, HeLa cells, MCF-7 cells, and human umbilical mesenchymal stem cells (HUMSC) treated by AuNP@CCM. (**E**) Expression levels of integrin α_v_β_3_ by flow cytometry and Western blot. (**F**) Annotated DFM images and count of plasmonic spots of HeLa cells and HUMSC pretreated with siRNA or cilengitide. Scale bar = 10 μm [96]. Copyright 2020, American Chemical Society.

Many reports have confirmed that integrins typically bind to the ECM [92]. The integrins α_V_β_3_ are expressed in cancer cells higher than normal cells [93], and integrin subunits α_3_, α_5_, α_6_, α_V_, β_1_ and β_3_, in particular, have been observed high expression in migrate cells [94]. Based on this, integrins are not only often used as a specific binding site for tumor-targeted drug delivery [95] but also widely believed to play a role in tumor migration rather than homologous targeting. However, some studies have also shown that integrins on cell surfaces can also serve as homologous target sites for cell-membrane-modified particles. For instance, Xie *et al.* investigated the cell uptake of cell-membrane-camouflaged nanoparticles (CMC-NPs) by a plasmonic imaging method (based on a dark-field microscopy (DFM)) and they found that cancer cell membrane-coated gold nanoparticle (AuNP@CCM) exhibited significantly higher cellular uptake than AuNP@Lipo, AuNP@RBC and AuNPs coated with trypsin-treated cancer cell membrane (AuNP@CCM^tryspin^) (Figure 3A). Combining to results of the co-culture with different inhibitors, they verified that integrin-mediated adhesion played an important role in homologous targeting of membrane-based systems (Figure 3B and C). In addition, their further results showed that high expression of integrin α_V_β_3_ on cancer cell membrane contributed to the cell uptake of CMC-NPs, whereas when expression of integrin α_V_β_3_ was down-regulated, this homologous-targeting uptake was significantly reduced (Figure 3D–F) [96].

Cadherin has been extensively studied in cancer biology including metastasis and tumor targeting. Unlike integrins that mainly promote cell adhesion to ECM, cadherins contribute mainly to homotypic cell–cell adhesion [79]. Cadherin is an abbreviation of ‘calcium-dependent adherent protein’, and they are divided into P-, E-, N-cadherins, etc., according to the tissues in which they were early found (i.e. placental, epithelial, nervous tissue). Also, cadherins are expressed in tumor cells and change in expression level during the process of epithelial–mesenchymal transition, i.e. a decrease in the expression of E-cadherins and an increase in N- or P-cadherins [97]. However, Padmanaban *et al.* also found that cells that had reduced protein levels of E-cadherins did not metastasize *in vivo* although they exhibited greater cell migration in culture *in vitro* [98]. This result increases the complexity of cadherins in the direction of tumor cell adhesion and targeting.

IgSF is the biggest group of CAMs that can form homophilic interactions with each other or heterophilic interactions with other nectins or other ligands [88]. For example, activated leukocyte cell adhesion molecule (ALCAM/CD166), a member of the IgSF, belongs to a recent subgroup with five extracellular immunoglobulin-like domains (VVC2C2C2). ALCAM mediates both heterophilic (ALCAM-CD6) and homophilic (ALCAM-ALCAM) cell–cell interactions [99]. In addition, IgSF is proven to form cell–cell adhesion between different cell adhesion systems (e.g. E-cadherin and nectin) [100]. Recent studies suggest that some IgSF is up-regulated in various cancers such as breast and lung cancers [101–103]. The relevance of IgSF to tumor immune response has been studied. CD47, for instance, one of the most well-known members of the IgSF, is overexpressed in cancer cells and wildly considered as a ‘don’t eat me’ signal [104]. Though the overexpression of IgSF proteins in cancer cells supports them as anti-cancer targets [105], the role of various IgSF in homologous targeting has not been known yet.

## Applications of homologous-targeting delivery systems on cancer therapy

**Figure 4.**
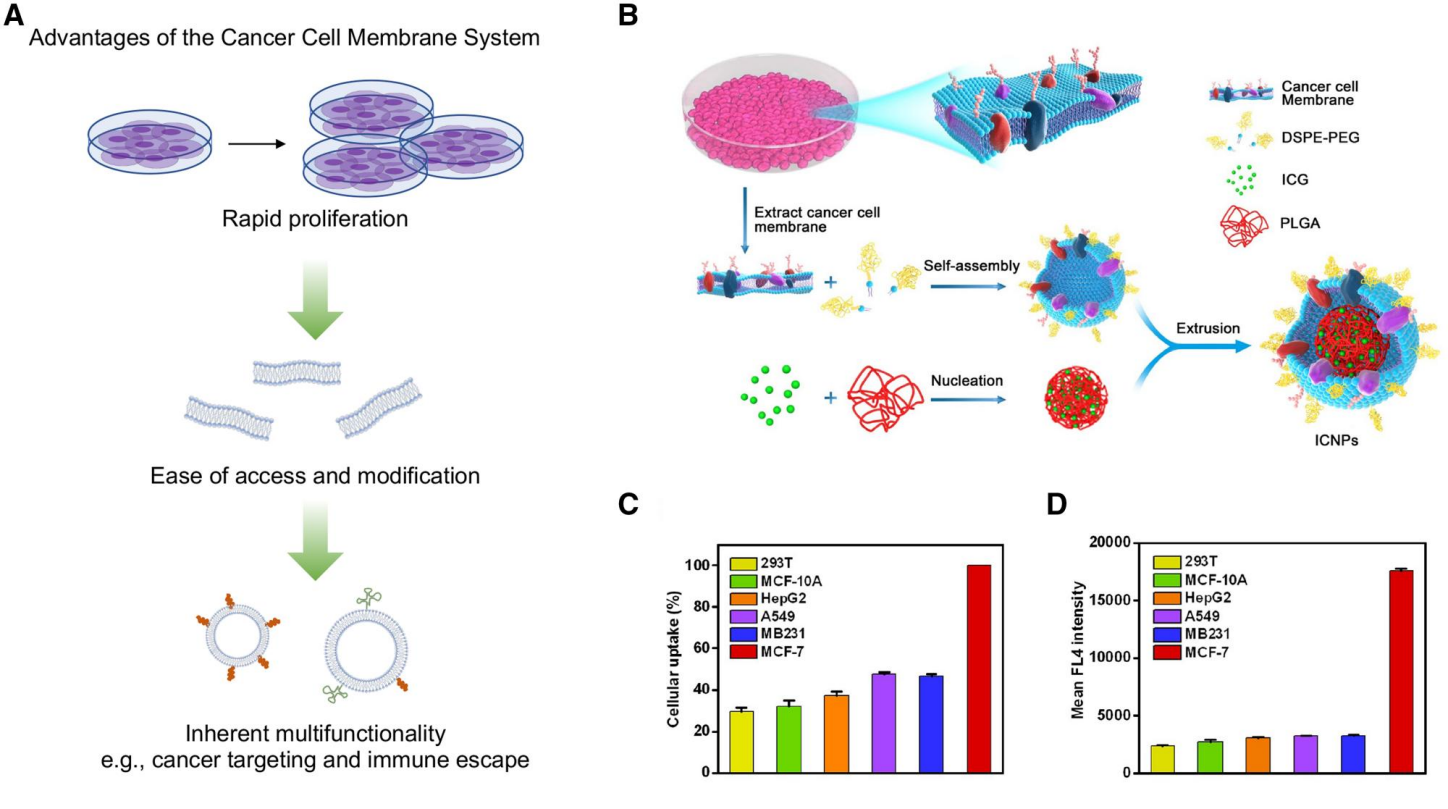
Illustration of the advantages of cell membrane-biomimetic nanoparticles and their application on targeting recognition of source cancer cells. (**A**) Main advantages of the cancer cell membrane system. (**B**) Illustration of preparation procedure of MCF-7 cell membrane−biomimetic nanoparticles. (**C**) Cellular uptake of MCF-7 cell membrane−biomimetic nanoparticles by different cells. (**D**) Mean fluorescence intensity of different cells after co-culturing with MCF-7 cell membrane−biomimetic nanoparticles [54]. Copyright 2016, American Chemical Society.

Basing on the homologous-targeting mechanism, various cancer cell- and cancer cell membrane-based systems have exhibited great progress. As summarized in Table 1, the use of a single-type cancer cell membrane or the whole cell has been able to achieve excellent homologous-targeting results. Sometimes, these systems are further modified on the surface (Figure 4A) or hybridized with different types of artificial or natural membranes (Table 2) in order to improve the performance of the carrier and enhance the effectiveness of cancer therapy. In this section, we present the composition of homologous-targeting systems in terms of pure cancer cell membranes, hybridization of cancer cell membranes with other membranes, and whole cancer cell to classify their development in the field of anti-cancer.

**Table 1.** The anti-cancer applications of homologous-targeting systems constructed by only cancer cell membranes or cancer cells

Types	Cancer cell lines	Guests	Loading methods	Applications	Ref.
Cancer cell membrane	HepG2	Pt(IV)-polymer nanoparticle	Extrusion	Targeted eradication of circulating tumor cells in whole blood by chemotherapy	[43]
	MDA-MB-231	Ru polypyridyl complex	Ultrasound at 37 kHz for 2 h and then extrusion	Cellular immune response enhancement and production of inflammatory cytokines including TNF-α, IL-12 and IL-6	[106]
	4T1	TPPa-Sy PLGA nanoparticle	Extrusion	TPPa-initiated OXPHOS inhibition in combination with the Sy-triggered glycolysis inhibition, resulting in lethal energy depletion	[107]
	B16F10	Dox@PMOF	Extrusion	ONOO^−^-based CDT and chemotherapy	[108]
	4T1	Dox loaded COF	Sonication and self-assembly	CDT and chemotherapy	[109]
	CT26	Gambogic acid-loaded PLGA nanoparticle	Extrusion	Chemotherapy and immunotherapy of CRC by regulating tumor immune microenvironment	[110]
	U14	Dexamethasone-loaded PLGA nanoparticle	Extrusion	Efficient modification of the TME to enhance the effects of gynecologic cancer chemotherapy	[111]
Cancer cell	A549	pCas9/gCDK4-encapsulated lipofectamine	Electrostatic interactions between cationic liposomes and membrane phospholipids	CRISPR-Cas9 editing-mediated CDK4 ablation, triggering synthetic lethal in KRAS-mutant NSCLC	[112]
	C1498	Dox	DNA intercalation and the electrostatic interactions between Dox and cytoplasm proteins	Chemotherapy and promotion of anti-tumor immune responses	[72]
	4T1	Dox-loaded liposome and αPD-1	Reaction with thiol groups between cell membrane and SPDP-modified αPD-1 and liposome with DSPE-PEG2000-MAL	Accumulation in pulmonary tumor and inhibition of lung metastatic 4T1	[74]

Ac4GalNAz, N-azidoacetylgalactosamine-tetraacylated; BDVs, bacteria-derived vesicles; CDK4, cyclin-dependent kinase 4; CDT, chemodynamic therapy; cGAMP, cyclic guanosine monophosphate-adenosine monophosphate; COF, covalent organic framework; DBCO-PEG4-NHS ester, dibenzocyclooctyne-PEG4-N-hydroxysuccinimidyl ester; DOX@PMOF, polymeric l-arginine polymer-coated doxorubicin-loaded metal-organic frameworks; DSPE-PEG2000-MAL, 1,2-dis-tearoyl-sn-glycero-3-phospho ethanolamine-N-[maleimide(polyethylene glycol)-2000]; NSCLC, non–small cell lung cancer; ONOO^−^, peroxynitrite; OXPHOS, oxidative phosphorylation; PLGA, poly(lactic-co-glycolic) acid; Ru, ruthenium; SPDP, N-succinimidyl-3-(2-pyridyldithio)propionate; Sy, syrosingopine; TME, tumor microenvironment; TPPa, triphenylphosphine-pyropheophorbide a; U14, mouse cervical cancer cells;

**Table 2.** The anti-cancer membrane-based systems constructed by hybridizing cancer cell membranes with other membranes

Membrane sources hybridized with cancer cell membranes	Cancer cell membrane sources	Guests	Applications	Ref.
Artificial lipids	SW1990, DPPC, DOPC, and DSPC	ICG, Dox	PTT and chemotherapy	[119]
	Metastatic melanoma cell and NBD-PE or Liss Rhod PE	Cob and Lenva	Improving cellular uptake and drug efficacy	[120]
	4T1, DOPC and DSPE-PEG_2000_	KTZ, BMS-202, ICG	PTT and immunotherapy	[121]
Erythrocyte	MCF-7 and RBC	Melanin NPs	PTT	[65]
	HCT116 and RBC	Oxaliplatin and Ce6	Synergistic Chemotherapy and PDT	[122]
	4T1 and RBC	Dox-loaded chitosan NPs	Chemotherapy	[123]
	HOS and RBC	Fe_3_O_4_ loaded PLGA NPs	Promoting the recruitment of macrophages and inducing macrophage polarization from M2 to M1, and achieving a ferroptotic and photodynamic synergistic therapy	[124]
Platelet	MDA-MB-231 and platelet	miRNA	The delivered anti-miRNAs are sensitized TNBCs to Dox	[125]
	Huh-7 and platelet	SFN and TPL-loaded LCNPs	promoting tumor cell apoptosis, inhibiting tumor growth, and achieving a better “synergy and attenuation effect” on hepatocellular carcinoma treatment	[126]
Peritumoral cells or relative cells	Glioma cells and GASCs	Temozolomide-encased PLGA NPs	Chemotherapy	[127]
	Glioma cells (U87-MG) and brain metastatic MCF-7	GA/ICG-loaded NPs	Endowing a capability of blood–brain barrier crossing owing to brain metastatic MCF-7	[128]
Leukocyte	4T1 and macrophage RAW 264.7	HPPH-loaded HSA NPs	Sonodynamic therapy	[64]
	4T1 and macrophage RAW 264.7	ICG, R837 and Fe_3_O_4_ NPs	CDT, immunotherapy and PTT	[129]
	4T1 and BMDCs	R837-loaded MSNs	Promoting CD8^+^ T cell-mediated immune response and modulating the tumor immunosuppressive microenvironment	[130]
	MCF-7	Fe_3_O_4_ NPs	Specific recognition and extraction of homologous CTCs	[59]
	4T1 and M1 macrophage (J774a.1)	R837-loaded PLGA NPs	Promoting immune response	[131]
Bacteria	4T1 and *E. coli*	PLGA NPs	Achieving individualized autologous tumor antigen vaccines	[132]
	4T1 and *E. coli*	Glutathione-decorated Te nanoparticles	Radiation-driven immunotherapy	[133]
Thylakoid	4T1 and thylakoid membrane	Tm_2_O_3_ NPs	NIR-stimulated ROS generation benefited from 3H4 state of Tm ions	[134]
Mitochondria	U87 MG and mitochondria	Gboxin-loaded PEG-PHB NPs	ROS-responsive Gboxin release, thus inducing a decrease of mitochondrial membrane potential and ultimately structural damage	[135]
Multiple membranes	BMDCs, DCs (B16), and *E. coli*	ONc-loaded F127 micelles	Enhanced accumulation in lymph nodes, immunological adjuvant, and tumor antigens	[136]

BMDCs, bone marrow-derived dendritic cells; CDT, chemodynamic therapy; Ce6, chlorin e6; Cob, cobimetinib; DC, dendritic cells; DOPC, 1,2-dioleoyl-sn-glycero-3-phosphocholine; Dox, doxorubicin; DPPC, 1,2-dipalmitoyl-sn-glycero-3-phosphocholine; DSPC, 1,2-distearoyl-sn-glycero-3-phosphocholine; GA, gambogic acid; GASCs, glioma-associated stromal cells; HOS, human osteosarcoma cell; KTZ, ketoconazole; LCNPs, lyotropic liquid crystalline lipid nanoparticles; Lenva, lenvatinib; Liss Rhod PE, 1,2-dipalmitoyl-sn-glycero-3-phosphoethanolamine-N-(lissamine rhodamine B sulfonyl); MSNs, mesoporous silica nanoparticles; NBD-PE, 1,2-dipalmitoyl-sn-glycero-3-phosphoethanolamine-N-(7-nitro-2-1,3-benzoxadiazol-4-yl) (ammonium salt); ONc, 5,9,14,18,23,27,32,36-octabutoxy-2,3-naphthalocyanine; PEG-PHB, poly (ethylene glycol)-poly (4-(4, 4, 5, 5-tetramethyltetramethyl-1,3, 2-dioxaborolan-2-yl) benzyl acrylate); PTT, photothermal therapy; R837, imiquimod; RBC, red blood cell; SFN, sorafenib; Tm_2_O_3_, thulium oxide; TPL, triptolide.

### Cancer cell membrane

As mentioned in Cancer cell membrane-based systems, cancer cell membranes are relatively easy to obtain, so homologous-targeting anti-cancer research based on cancer cell membranes is more extensive [33]. Currently, cancer cell membranes have been used for homologous-targeting delivery of a variety of substances and have proven to dramatically increase the rate of homologous-targeting delivery of substances [113, 114]. Chen *et al.* employed breast cancer human breast cancer cells (MCF-7) cell membranes to coat indocyanine green (ICG)-loaded PLGA nanoparticles and their results demonstrated that homologous cells had significantly higher cellular uptake of MCF-7 cell membrane-coated nanoparticles than other cells (Figure 4B). By detecting the fluorescence intensity of the drug inside the cells, they found after coating with MCF-7 cell membrane, the drug delivery rate was also significantly higher in MCF-7 cells than other cells, including normal cells and other cancer cells (Figure 4C and D) [54]. Wu *et al.* mixed cultures of different cancer cell membrane vesicles with one cancer cell, followed by fluorescence-activated cell sorter analysis, and the results also confirmed homology of cancer cellular uptake [115].

**Figure 5.**
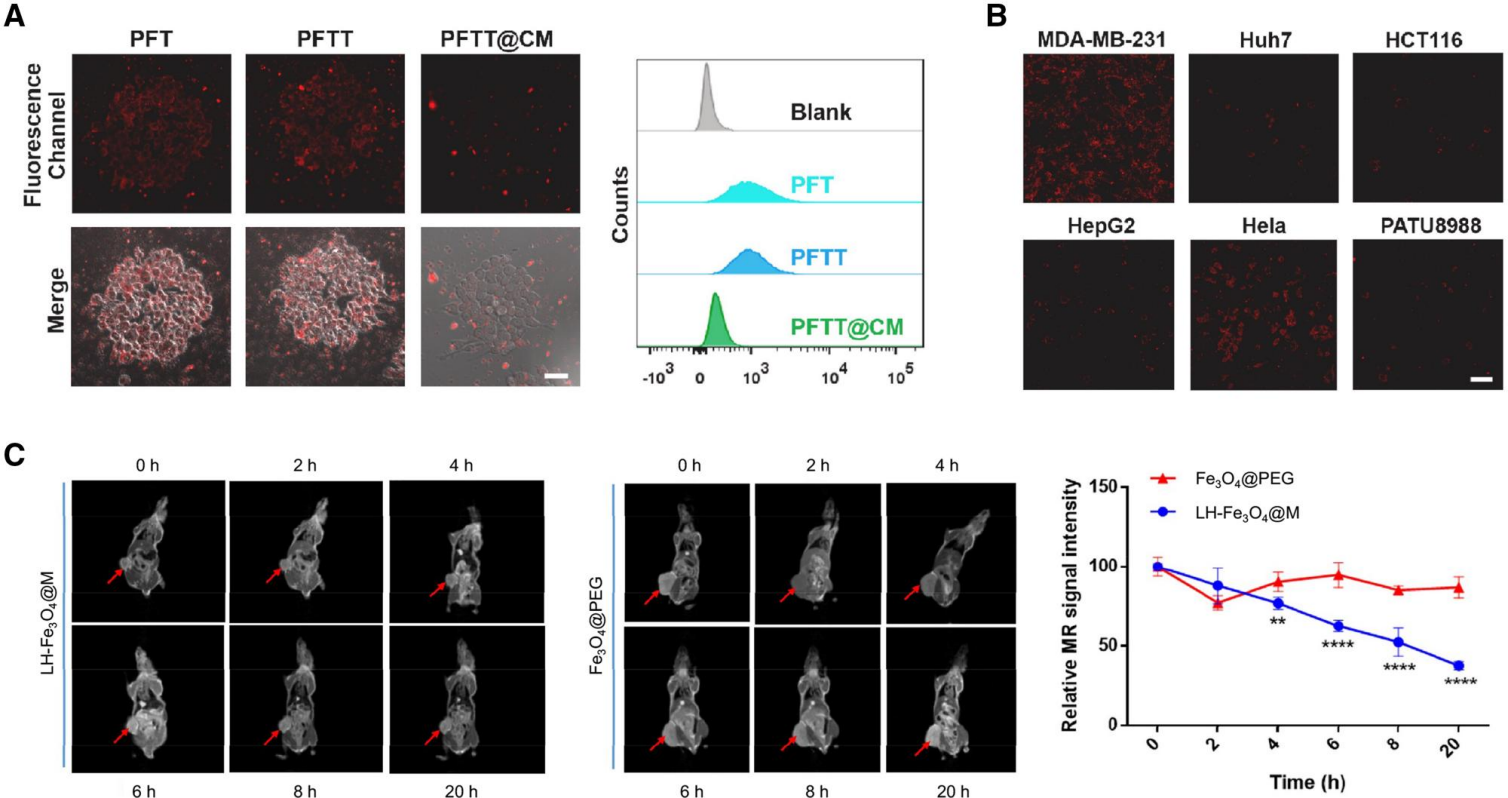
The immune escape and homologous selectivity of cancer cell membrane-based systems. (**A**) Confocal laser scanning microscope (CLSM) images and flow cytometry analysis of RAW 264.7 after the treatment of PFT, PFTT and PFTT@CM, respectively. Scale bar = 20 μm. (**B**) CLSM images of several cells after being treated with PFTT@CM, respectively. Scale bar = 20 μm [58]. Copyright 2022, Elsevier Ltd. (**C**) Magnetic resonance imaging and relative tumor MR signal intensity of mice after treatments of the membrane-coated magnetic nanoparticles Fe_3_O_4_@M-LH and the magnetic nanoparticles Fe_3_O_4_@PEG at different time points, respectively (***P < 0.001, ****P < 0.0001) [117]. Copyright 2023, the Authors, under exclusive license to Springer Science Business Media, LLC, part of Springer Nature.

The delivered guests of the cancer cell membrane-based system include virtually all substances that can be used in cancer therapy such as metal ions [56], small molecules (e.g. chemotherapeutic drugs, photosensitizers, etc.), RNA (siRNA, shRNA and miRNA) [116], peptides, proteins and functionalized nanoparticles. For example, Pan *et al.* synthesized nanoscale PVP dispersed Fe-TCPP and coated it by breast cancer cell (MDA-MB-231) membranes for highly specific and efficient tumor therapy [58]. Their results showed that the membrane-coated nanoparticles PFTT@CM possessed better *in vitro* anti-immune clearance and high specific selectivity than the nanoparticles PFT and PFTT without membrane coated. As shown in Figure 5A and B, the obtained membrane-coated nanoparticles showed significantly low cellular uptake of RAW 264.7 cell line and high internalization in homologous cells MDA-MB-231 compared to other cells, including Huh7, HCT116, HepG2, Hela, and PATU8988 [58]. Meanwhile, guests can be multiple substances. In the work of Li *et al.*, lycorine hydrochloride and magnetic iron oxide simultaneously encapsulated into nanosystems by CRC cell membrane (Fe_3_O_4_@M-LH). The homologous-targeting property enhanced not only chemotherapy but also magnetic resonance imaging efficiency of Fe_3_O_4_@M-LH (Figure 5C) [117].

It is worth noting that these delivered guests can not only be encapsulated inside the membrane-based system but also modified outside the membrane. For instance, Liu *et al.* modified the 3' end of functional DNA with cholesterol to allow anchoring of DNA on the surface of melanoma cell membrane-based systems, thereby achieving specific recognition in specific cells [52]. In this way, one membrane system can obtain more opportunities to achieve multiple functions.

### Artificial hybridized membrane systems

Although an individual cancer cell membrane is capable of good homologous targeting, there are some drawbacks such as lack of further functionalized modifications and limited immune escape ability [65, 118]. In order to alter the physicochemical properties (e.g. fluidity, surface potential, indication of specific group modifications, etc.) and improve the delivery efficiency of cell membrane systems, researchers hybridize cancer cell membranes with functional artificial lipids or other membranes, like platelet, erythrocyte, leukocyte and bacteria membrane (Table 2). This hybridization approach is usually easy to endow membrane systems with more functionality while circumventing the difficulty of modifying membranes by some physicochemical methods.

#### Hybridization of cancer cell membranes and artificial lipids

Hybridization of cancer cell membranes with artificial lipids such as conventional liposomes, emerges relatively early. This approach can confer immune escape and tumor homologous targeting capabilities to liposomes based on the use of small amounts of cancer cell membranes. For instance, Su *et al.* hybridized the membrane of human pancreatic cancer cells (SW1990) and drug-carrying liposomes at a weight ratio of 1:100, allowing membrane proteins to be modified easily on the surface of liposomes while maintaining stable drug loading [119]. The resulting hybrid membrane system exhibited a significantly high *in vitro* uptake of SW1990 cells. *In vivo* tumor enrichment of the drug was detected by fluorescence, and the hybridized membrane system showed nearly twice the tumor enrichment of the regular liposomes 24 h after treatment [119].

Moreover, the physicochemical properties of the membrane system can be easily modulated due to the presence of artificial lipids [137]. Huang *et al.* found that addition of artificial lipid to cell membranes could regulate the fluidity and permeability of the membrane, reducing exposure of inside delivered guests [138]. It has also been shown in many studies that the use of anionic or cationic lipids significantly modulates the *in vivo* organ distribution of liposomes and that the modulation of the degree of rigidity or softness of the membrane system by lipid portions also plays a key role in the *in vivo* distribution [139]. Theoretically, cancer cell membrane systems hybridized with artificial lipids can easily regulate the primary targeting of *in vivo* distribution through physicochemical properties, and then secondary targeting through homologous adhesion to cancer cell membranes, improving enrichment and therapeutic efficiency. Yet whether the above two factors have a good stacking effect still needs to be further confirmed.

#### Hybridization of cancer cell membranes and other cells

Recently more studies tend to construct hybridized membrane systems of cancer cells with other cells such as platelets, erythrocytes, stem cells, immune cells, etc. Although cancer cells also express some proteins like CD44 and CD47 on their membranes [140], cancer cell membrane systems exhibit a limited immune escape in some instances due to the differences in the expression of these proteins in different types of cancer cells and at different stages of development, so that hybridizing them with other cells can result in better and stable mimetic properties, i.e. safety, biocompatibility and immune escape. In addition, a variety of cell membranes provide different functions as well. For instance, erythrocyte and platelet membrane-based systems evade immune clearance because of the presence of the surface CD47 protein inherited from the source cells, significantly prolonging their blood circulation [122, 141]. Zheng *et al.* reported that RBC membrane was fused with cancer cell membrane derived from human colon cancer cells HCT116 to fabricate an erythrocyte-cancer cell hybrid membrane and then compared the efficiency of phagocytosis of the hybrid membrane nanoparticles by macrophages with that of only HCT116-membrane nanoparticles, showing that the presence of RBC membranes significantly reduced immune clearance [122].

**Figure 6.**
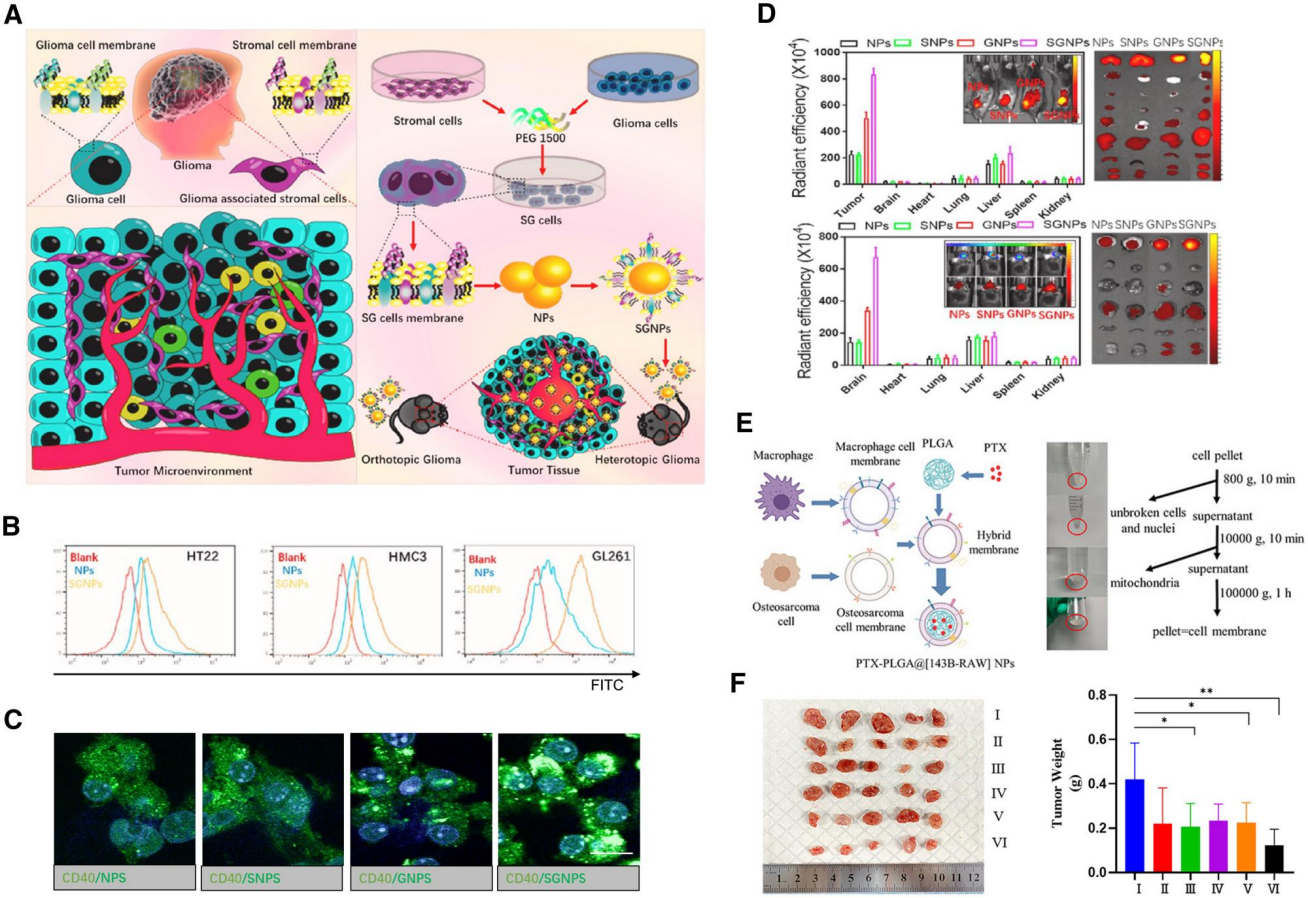
Applications of hybrid systems of cancer cell membranes and other membranes on cancer therapies. (**A**) Illustration of TME drug delivery system SGNPs for targeted chemotherapy of gliomas. (**B**) Cellular uptake of SGNPs by neuron cells (HT22), astrocyte (HMC3), and mouse gliomas cells (GL261). (**C**) CLSM images of cellular internalization of C6-loaded NPs. (**D**) Biodistribution of IR-780-loaded NPs in the tumors and main organs from heterotopic glioma models and orthotopic glioma models [127]. Copyright 2023, Elsevier Ltd. (**E**) Illustration of preparation of hybrid membrane-coated NPs and images showing membrane extraction process. (**F**) *In vivo* anti-tumor effect of various samples (I: PBS, II: Free PTX, III: PLGA-PTX NPs, IV: PTX-PLGA@RAW NPs, V: PTX-PLGA@143B NPs, VI: PTX-PLGA@[143B-RAW] NPs). (*P < 0.05, **P < 0.01) [143]. Copyright 2021, Dove Medical Press Ltd.

To further enhance the targeting effect, Ma *et al.* isolated GASCs and glioma cells from a clinic and constructed hybrid membrane (stromal cell-glioma cell fusion cell membrane)-coated nanoparticles (SGNPs) that exhibited a higher *in vitro* cellular uptake and *in vivo* biodistribution than mono-membrane-coated nanoparticles (either GNPs or SNPs) due to the enhanced efficiency of tumor microenvironment targeting that was contributed to the existence of GASCs membrane (Figure 6A–D) [127]. These results prospect that combining with the peritumoral cells or cancer-associated stromal cells may promote the tumor targeting delivery for guests and the development of a multi-layer target.

Macrophages, a type of leukocyte abundantly present in the tumor microenvironment, are frequently utilized in biomimetic DDSs, which have been proven the capabilities of infiltrating and penetrating deep into tumor tissue [142, 143]. Chen *et al.* revealed that α4-integrins on macrophages tethered to VCAM-1 on the surface of cancer cells, enabling active targeting, especially of metastatic tumor cells [50, 51]. Moreover, macrophage membrane systems exhibited good immune escape. The results of Xuan *et al.* demonstrated that macrophage membrane-coated MSNs remained more than 30% free after co-incubation with macrophages for 24 h, whereas MSNs without macrophage membrane coating were completely phagocytosed [144]. However, as the binding of macrophages to cancer cells is not homologous, it may have the potential risk of off-targeting in clinically different individuals compared to homologous targeting of cancer cell membranes. The hybrid membrane system of the macrophages and cancer cells can play well with the strengths of both and compensate for each other's weaknesses. Cai *et al.* coated the paclitaxel-loaded PLGA nanoparticles with a hybrid membrane from human osteosarcoma cell 143B and mouse mononuclear macrophage RAW 264.7 [143]. Their results demonstrated that PLGA nanoparticles with RAW 264.7 membrane modification alone did not have good cellular uptake, whereas PLGA nanoparticles with 143B membrane modification (either cancer cell membrane alone or hybridized membranes of cancer cells and macrophages) showed excellent homologous cancer cell uptake. Similarly, hybridized membrane-coated PLGA nanoparticles had a higher *in vivo* tumor enrichment and a better anti-tumor effect (Figure 6E and F) [144]. Ji *et al.* isolated and constructed cancer cell-macrophage hybrid membranes to coat hollow copper sulfide (CuS) NPs, where macrophage and cancer cell membranes contributed by RAW 264.7 and murine hepatic cancer cells (H22), respectively [145]. They found that biomimetic hybridized nanoparticles had significantly lower cellular uptake of non-homologous cancer cells (human monocytic leukemia, THP-1) compared to nanoparticles without cell membrane coating, which suggests that the modification of cancer cell membranes does not broadly enhance uptake by cancer cells, but rather confers a specific selectivity for homologous cells. Interestingly, by co-incubating with human hepatic cancer cells (HepG2, the cell line is not homologous with H22 although they are the same type of cancer), biomimetic hybridized nanoparticles still showed a significantly higher uptake rate compared to nanoparticles without cell membrane coating [145]. The authors suggested that macrophage membranes played a role in this process. Similar results can be seen in the work of Zheng *et al.* who found that the human-derived HCT116 colon cancer cell membrane system showed high *in vitro* uptake in both HCT116 and CT26 (colon cancer cells), but homologous HCT116 exhibited relatively higher uptake [122]. These results imply that homologous targeting may be involved by substances with commonalities in the same type of tumor, rather than absolute homologous tumor, as discussed previously for thepotential mechanisms of homologous adhesion and targeting . Notably, however, this homotypic but non-homologous tumor-targeting advantage disappeared *in vivo* [122].

Additionally, lymphocyte function-associated antigen-1 is expressed on some leukocytes (e.g. lymphocytes, monocytes granulocytes, etc.) membranes and specifically binds to intercellular cell adhesion molecule-1 that usually highly expressed in tumor-associated blood vessels, thereby increasing leukocyte permeability to tumor blood vessels [146, 147]. Therefore, the lymphocyte-cancer hybridized membrane systems are allowed to be used for a multistage delivery (blood circulation—tumor tissue—tumor cells), enabling high targeting and low-toxicity therapies.

Leukocyte-cancer hybrid cell membrane systems are often applied to specific adhesion and separation of CTCs *in vitro* [59, 148]. For example, Ding *et al.* employed membranes of MCF-7 and RAW 264.7 to construct hybridized membrane systems, thereby achieving both targeting ability and immunological surveillance [148]. Stemming from the above inspiration, we believe that hybrid-cell-membrane-based biomimetic systems will have great potential in treating not only CTCs but also metastatic tumors.

In addition to the membrane from erythrocytes and immune cells, stem cells are used earlier for drug delivery into tumor cells because of their capability of self-renewal, relative ease of isolation and *in vitro* expansion, and homing capacity [149, 150]. Zheng *et al.* constructed a hybrid membrane system using MSC membranes and pH-sensitive liposomes, achieving tumor target and escape from endo/lysosomes after endocytosis, respectively [66]. Meanwhile, research has identified that the low efficiencies in some cancer such as CRC and head and neck squamous cell carcinoma (HNSCC) contributed to cancer stem cells (CSCs) that promote tumor relapse or diaspora of the original tumor and existence in the invasive area of the tumor sites [151–153]. Unfortunately, there is a lack of strategies that can target CSCs. Bu *et al.* proved a high anti-tumor efficiency of a CSC-membrane system in the immunocompetent Tgfbr1/Pten 2cKO HNSCC mouse model, which contains a more complex tumor microenvironment that is more similar to the human HNSCC microenvironment [151]. They indicated that the CSC membrane system that carried both cancer cell markers and CSC markers could offer enhanced CSC targeting ability [151]. We believe that it is for this reason that CSCs usually no longer construct hybrid systems with cancer cell membranes.

#### Hybrid membrane of cancer cells and bacteria

**Figure 7.**
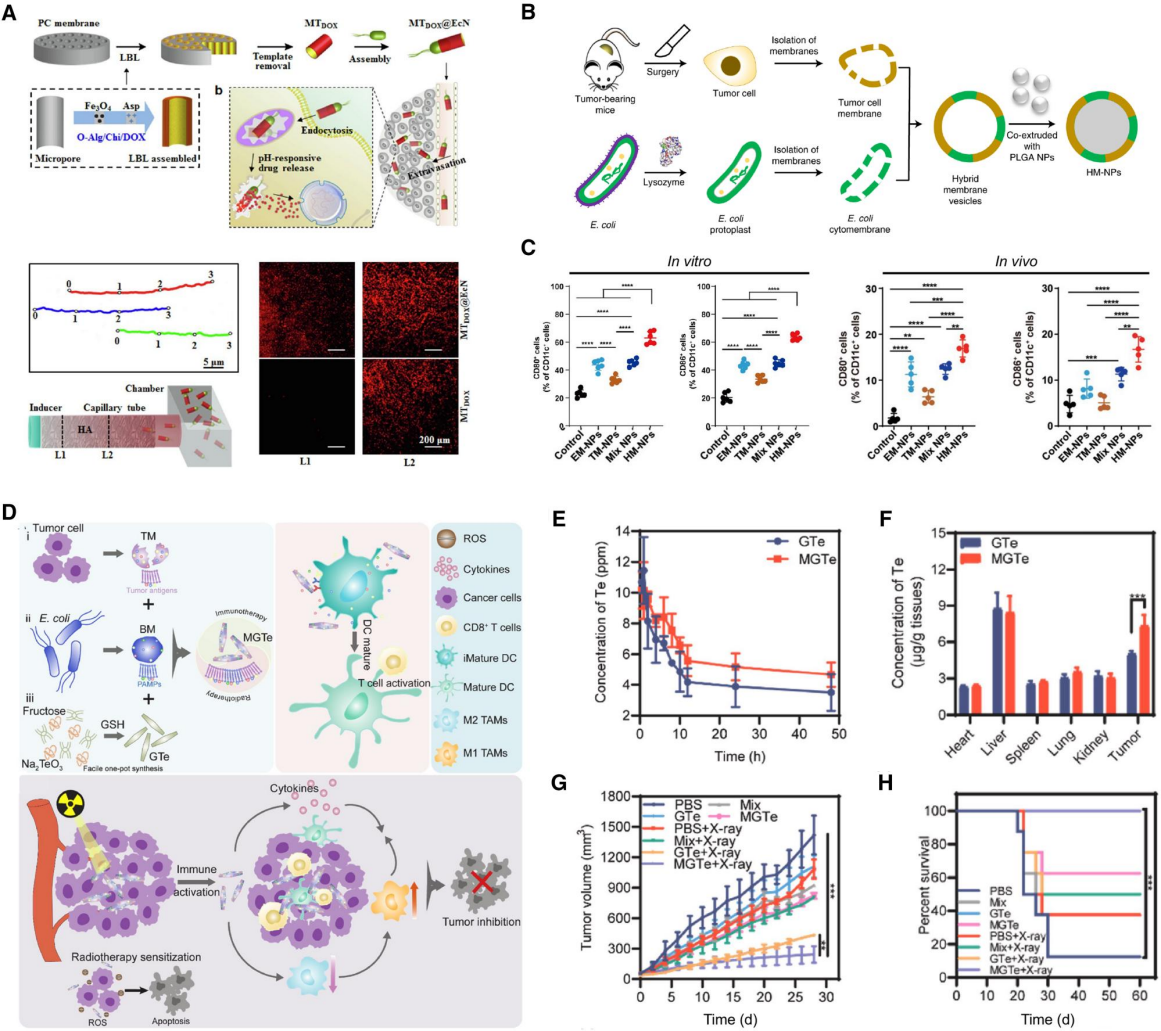
Bacteria and bacteria-based hybrid membrane systems for cancer therapies. (**A**) Illustration of the preparation process and motility of the microrockets [157]. Copyright 2019, Elsevier B.V. (**B**) Illustration of preparation of HM-NPs [159]. Copyright 2022, Springer Nature Limited. (**C**) *In vitro* and *in vivo* expression of CD80 and CD86 suggesting HM-NPs with the capacity to promote DC maturation [132]. Copyright 2021, the American Association for the Advancement of Science. (**D**) Illustration of MGTe to realize synergistic radiotherapy sensitization and immunotherapy enhancement for breast cancer eradication. (**E**) Time-dependent Te element analysis by ICP-MS in plasma within 48 h after GTe or MGTe intravenous injection. (**F**) Biodistribution of Te element after 48-h intravenous administration with GTe or MGTe. (**G**) Tumor volumes of 4T1-bearing mice with different treatments. (**H**) Survival curves of unilateral 4T1-bearing mice after various treatments [133]. Copyright 2022, Elsevier Ltd.

Microorganisms-based drug carriers including outer membrane vesicles, bacterial ghosts, and archaeosomes have been utilized in cancer treatments due to their properties [154]. For instance, *C. novyi-NT* achieved Dox concentration in tumor sites over twice higher than that of Doxil alone due to the degradation of the ECM of collagen by bacterial proteolytic activity, which further increased convection through vessel walls [155]; *Escherichia coli* (*E. Coli*) tend by a concentration gradient of L-aspartate, in which it moves from lower to higher gradient [156]. Xie *et al.* constructed a microrocket by *E. coli* Nissle1917 (EcN) and achieved a directed motion of the microroket in both water and viscous media (Figure 7A), resulting in a 6.1-fold higher tumor enrichment of Dox compared to a delivery system without an EcN component [157].

Meanwhile, bacteria outer membrane (BM) has been considered as a natural immune adjuvant and designed as a cancer vaccine [132, 158], which could overcome the immune-suppressing barriers to activate antitumor T-cell response preferably, resulting in efficiently innate and adaptive immunity for tumor-specific antigens presentation [159]. Nie and his team fused the bacterial cytoplasmic membrane and the primary tumor cell membrane from surgically removed tumor tissues to construct hybrid systems as personalized cancer vaccines (Figure 7B) [159]. Their further research showed in the hybrid systems, tumor cell membranes from resected autologous tumor tissue provided the immunogenicity of autologous tumor antigens and *E. coli* cytoplasmic membranes induced dendritic cell maturation *in vitro* and *in vivo*, activating splenic T cells (Figure 7C) [132]. Thus, by combining the hybrid membrane system of bacterial and tumor membranes, the system delivers other components for tumor diagnosis and treatment. For example, Wang *et al.* encapsulated polydopamine in the hybrid membrane system for homologous targeting followed by tumor photothermal therapy [160]; Pan *et al.* employed the hybrid membrane system to deliver glutathione (GSH)-decorated Te nanoparticles (MGTe). By comparing to GSH-decorated Te nanoparticles without membrane coating (GTe), they found that MGTe enabled long circulation and tumor enrichment of Te nanoparticles to further improve tumor radiotherapy efficacy (Figure 7D–H) [133].

### Cancer cells as carriers

Anti-tumor strategies using intact or engineered cells for substance delivery have also progressed [74]. Fang and his team used live macrophages to adhere to a large number of Dox-containing nanoparticles for their immune escape and tumor enrichment [161]. In their strategy, the drug-loaded nanoparticles adhered to the outside of the macrophage membrane instead of loading inside the cell, thus reducing the effect of the loading process and the loaded drug on macrophage activity [161]. Platelet is also used as a carrier for drug delivery though it was reported that the activated platelet can release TGF-β1 and VEGF that are possibly related to tumor metastasis [162, 163]. In addition, stem cells such as MSC and NSC have been investigated as DDSs for cancer therapy in clinical trials [164].

**Figure 8.**
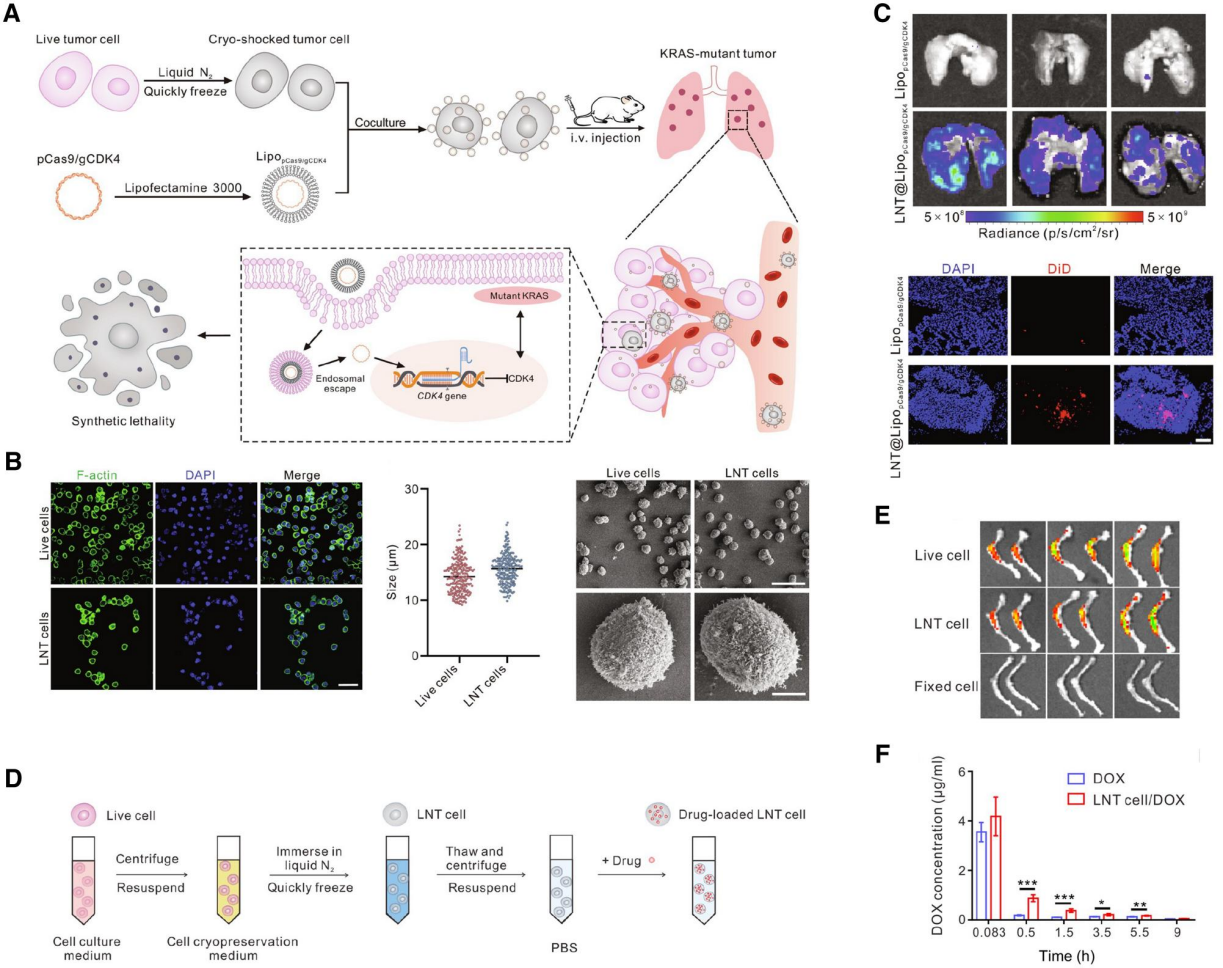
Cancer cells as carriers for homologous cancer therapy. (**A**) Illustration of preparation of cryo-shocked cells delivery system (defined as LNT) from A549 cells and its application in cancer treatment. (**B**) Representative cell structure images, size and SEM images of LNT and live A549 cells. Scale bar for CLSM image = 50 μm, and scale bar for SEM images = 50 μm (top) or 5 μm (bottom), respectively. (**C**) Fluorescence images of isolated lungs and CLMS images after treatment with DiD-labeled liposomes and liposome-loaded LNT for 6 h. Scale bar = 50 μm [112]. Distributed under a Creative Commons Attribution NonCommercial License 4.0 (CC by-NC). Copyright 2024, The American Association for the Advancement of Science. (**D**) Illustration of the procedure to prepare LNT cells from AML cells. (**E**) Fluorescence images of bone isolated 6 h after injection of cy5.5-labeled live C1498 cells, LNT C1498 cells, and paraformaldehyde-fixed C1498 cells. (**F**) Plasma Dox concentration after intravenous injection of free Dox and Dox-loaded LNT cell [72]. Distributed under a CC by-NC. Copyright 2020, The American Association for the Advancement of Science.

The engineered cancer cells have generally undergone special inactivation treatments that prevent them from possessing the proliferation ability while maintaining long circulatory retention, biocompatibility, easy surface modification and homologous targeting properties. Comparing to cell membrane-based delivery systems, cell, as carrier, has been proven better *in vitro* cytotoxicity, longer circulation time and higher tumor accumulation in RBC system [165]. This may be due to the fact that cell-based systems are more bionic and more difficult to be cleared rapidly by the immune system than cell membrane-based systems, which are smaller in size, have lower membrane integrity, and have mechanical properties that differ from those of the whole cells. However, to our knowledge, there are not many reports of such direct use of intact cancer cells as homologous-targeting vectors. Recently, Liu *et al.* employed cryo-shocked non-small cell lung cancer cells (A549) to target deliver CRISPR-Cas9-loaded liposome, achieving editing-mediated CDK4 ablation triggers synthetic lethal in KRAS-mutant non–small cell lung cancer and prolongs the survival of mice, where the cryo-shocked A549 cells preserved the original structure (Figure 8A and B) and enabled highly targeted lung delivery through either passive trapping by lung capillaries or preserved by glycoprotein (CD44) on A549 cell surface (Figure 8C) [112]. This method to obtain cryo-shocked cancer cells was reported in their previous work, in which AML cells were treated with liquid nitrogen to maintain homologous-targeting ability and delivered Dox to the bone marrow (Figure 8D–F) [72], which is also called the homing effect in myeloid leukemia. Similarly, Zhang and his team used a technique of tumor cell enucleation, resulting in tumor cells that lost their ability to proliferate and cause disease, but retained their basic cytoskeleton and subcellular structure, and retained important physiological functions as ‘living cells’, as well as possessing tumor cell antigens that are recognized by the immune system [166]. Although in this work this enucleated melanoma B16F10 cells, as a whole-cell cancer vaccine, carrying cyclic guanosine monophosphate-adenosine monophosphate-loaded bacteria-derived vesicles to boost tumor-specific T-cell infiltration [166], the cancer cells prepared by this enucleation technique are also suitable for preparation of homologous-targeting cancer cells. On the other hand, however, plenty of research demonstrated that inactivated or enucleated cancer cells not only retain tumor antigens to elicit vaccination effects but also tumor-targeting endothelial adhesive capabilities through overexpressing certain adhesion molecules such as CD44 and E-cadherin [98, 167], some concerns exist about nonspecific stimulation of the immune system that may elicit an undesirable immunopathological state and whether other substances in the ‘dead cells’ produces further positive effects or side effects deserves more in-depth exploration.

## Limitations of current homologous-targeting strategies

Despite significant progress in tumor therapy research, homologous-targeting delivery systems based on cancer cells or cancer cell membranes still face several limitations. Firstly, these systems rely on receptors and antigens on the surface of cancer cell membranes for targeting. However, tumors exhibit substantial heterogeneity, and the types and ratios of proteins on cancer cell membranes can vary significantly between patients or even within the same patient over time. This variability can reduce the effectiveness of tumor targeting. Additionally, the promising results seen *in vitro* and *in vivo* are often achieved using stable cell lines with consistent protein expression. In clinical settings, however, not all tumors are sufficiently specific or express consistent surface markers, making it challenging to ensure that tumor cell membrane-derived vectors provide the same targeting effect across different tumor types.

Secondly, despite the advantages of cancer cells and their membranes in terms of accessibility and homologous targeting, researchers sometimes prefer non-cancer cells as tumor-targeting delivery vehicles, such as stem cells, natural killer (NK) cells, macrophages, erythrocytes, neutrophils, and lymphocytes [168–171]. For example, Ping and his team developed a series of engineered immune cells for cancer immunotherapy [172, 173]. This preference may stem from biosafety concerns, as cancer cells contain many proto-oncogenes, and the extracellular vesicles they release can promote tumor growth and angiogenesis. Another reason could be that the mechanism behind homologous targeting in cancer cell- and membrane-based systems is not yet fully understood. It is generally believed that CAMs on cancer cell surfaces contribute to targeting. However, most CAMs are non-specific and are somewhat common across various cancer types. Nevertheless, numerous studies have shown that cancer cell membrane-based systems exhibit high cellular uptake and tumor enrichment specifically for homologous cancer cells [54, 115]. This suggests that additional or unidentified factors may be involved in the process of homologous targeting. It is also possible that homologous adhesion is regulated by a complex interplay of CAM components, with distinct proportions or activities in different cancer cells.

Additionally, while studies have compared the tumor-targeting efficiency of nanoparticles delivered via cell membrane-encapsulated nanoparticles versus intact cell-based systems [165], there is still a lack of systematic research on the stability of cancer cell- and cancer cell membrane-based systems. Due to their biological activity, these systems, especially those involving live cancer cells, may have lower stability during storage compared to other current delivery systems, whether organic, inorganic, or hybrid. It has been shown that in cell membrane-coated nanoparticle systems, the internal nanoparticles help maintain membrane stability and function [174, 175]. However, in the context of clinical precision therapy, this may not be a major concern. In conclusion, although cancer cell- and cancer cell membrane-based systems show promising homologous-targeting effects, further comprehensive studies are necessary to fully understand and optimize their potential.

## Summary, outlook and translational potential

DDSs based on cancer cells and cancer cell membranes have been extensively developed and widely explored for homologous targeting and tumor treatment. Currently, these systems primarily function in immune evasion and precise tumor targeting. Additionally, the use of cancer cell membrane components as cancer vaccines to enhance immune response has gained attention [176], suggesting that cancer cell-based systems can serve multiple roles, such as drug delivery and cancer vaccine adjuvants, offering more comprehensive and potent cancer therapies [177, 178]. These advantages and features highlight the great potential of cancer cell- and cancer cell membrane-based homologous-targeting strategies for clinical translation. However, further research is needed to determine whether these inactivated cells carry other endogenous substances during drug delivery, potentially leading to effects such as localized inflammation or tumor recruitment and migration.

Homologous targeting is a good strategy for the guests to efficiently cross many biological barriers and reach the tumor site with cancer cell- or cancer cell membrane-based systems. For the cancer cell-based systems, the tumor site is often their expected endpoint. If the delivered substances are small molecule drugs or macro-biomolecules (e.g. proteins and peptides), they can form an *in situ* high drug concentration to kill cancer cells. Whereas, if the delivered substances are nanoparticles, these nanoparticles are usually designed to be able to dissociate from the original cell-based carrier at the tumor site and enter the target cells through endocytosis [161]. For the cancer cell membrane-based systems, their function not only is homologous adhesion to cancer cells but also requires the guest to be internalized by target cells. It is still unclear whether the internalization of micro-/nano-size cancer membrane-based systems can be affected by their inherent properties. Recently, it has been reported that cellular uptake of liposome nanoparticles is not affected by the physicochemical properties of particles, including size, surface charge, and chemical modifications, but rather correlates with the expression of specific proteins in co-cultured cells [179]. Then the performance of cancer membrane-based systems, which have a bilayer structure similar to that of liposomes and are rich in various membrane proteins or other components, in corona adsorption and cellular uptake need to be further explored.

Currently, some cell membrane-based systems are in preclinical stages for tumor treatments [180]. The accessibility and straightforward fabrication of cancer cells or cancer cell membranes make them suitable for both research and clinical applications. In clinical settings, cancer cells and membranes can be isolated and extracted from a patient’s primary tumor, offering potential for personalized precision therapy. These processes could be somewhat analogous to chimeric antigen receptor (CAR) T-cell therapy [181], with the distinction that the extracted cells are cancer cells, not genetically modified *in vitro*. Instead, various cancer cell- and cell membrane-based systems are fabricated as needed for the patient, enabling homologous-targeting cancer therapy. Since cancer specificity varies from patient to patient and cancer cell characteristics differ, developing a universal agent applicable to all patients is challenging. Therefore, finding a rapid, automated, and mechanized process to reduce costs and improve efficiency is a crucial consideration for clinical translation.
